# Moderate tetrabasic zinc chloride supplementation improves growth performance and reduces diarrhea incidence in weaned pigs

**DOI:** 10.5713/ajas.18.0914

**Published:** 2019-05-27

**Authors:** Gang Zhang, Tian Xia, Jinbiao Zhao, Ling Liu, Pingli He, Shuai Zhang, Liying Zhang

**Affiliations:** 1 State Key Laboratory of Animal Nutrition, China Agricultural University, Beijing 100193, China

**Keywords:** Growth Performance, Weaned Pigs, Tetrabasic Zinc Chloride, Zinc Excretion

## Abstract

**Objective:**

Two experiments were conducted to evaluate tetrabasic zinc chloride (TBZC) on the health of weaned pigs, and to determine the optimal supplemental concentrations and whether dietary TBZC could replace the pharmacological concentrations of dietary zinc oxide (ZnO) to improve growth performance and decrease Zn excretion in weaned pigs.

**Methods:**

In Exp. 1, 180 weaned pigs (8.92±1.05 kg body weight [BW]) were randomly assigned to 1 of 5 treatments, including the basal diet containing 125 mg/kg zinc sulfate (ZnSO_4_), and the basal diet with 1,200, 1,800, 2,400, or 3,000 mg/kg TBZC supplementation. In Exp. 2, 240 weaned pigs (7.66±1.09 kg BW) were randomly assigned to 1 of 5 treatments, including a negative control diet without Zn supplementation, a positive control diet (2,250 mg/kg ZnO), and 3 experimental diets with different concentrations of TBZC supplementation (1,000, 1,250, and 1,500 mg/kg).

**Results:**

In Exp. 1, the average daily gain (ADG), feed efficiency (G:F) and diarrhea incidence responded quadratically (p<0.01) as the TBZC supplemental concentrations increased, and pigs fed 1,200 and 1,800 mg/kg TBZC showed the best growth performance. Moreover, 1,800 mg/kg TBZC supplementation showed the greatest (p<0.01) total antioxidant capacity and glutathione peroxidase activities in liver of pigs. Histopathological examination revealed lesions in heart, liver, lung and kidney, and mild or severe histological lesions mainly occurred with the supplementation of 2,400 and 3,000 mg/kg TBZC. In Exp. 2, 1,000 and 1,250 mg/kg TBZC supplementation in diets significantly (p<0.01) increased ADG and G:F of weaned pigs, reduced Zn excretion in feces, and had no effect on diarrhea-reducing compared to 2,250 mg/kg ZnO supplementation.

**Conclusion:**

The TBZC is a potential alternative to ZnO. The recommended concentration of TBZC in weaned pig diets is 1,000 to 1,250 mg/kg.

## INTRODUCTION

Post-weaning diarrhea is one of the most common causes of morbidity and mortality for weaning pigs, and hence greatly impairs their growth performance [1]. Many studies and practical experiences have shown that inclusion of pharmacological doses of zinc oxide (ZnO) in weaned pig diets can improve growth performance and decrease diarrhea incidence [2,3]. However, feeding high concentrations of ZnO (more than 2,250 mg/kg) to weaned pigs can result in environmental concerns due to excess zinc (Zn) excretion in the manure. Excess accumulation of Zn in soil could deteriorate the soil quality, produce toxicity to plants, and potentially impair the health of animals and humans [3–5]. The influence of Zn excretion on the environment is long-lasting, therefore, it is suggested to formulate lower concentrations of Zn in animal diets and supplement Zn with high bioavailability to decrease the its excretion in weaned pigs.

Tetrabasic zinc chloride (TBZC) is an inorganic form of Zn produced by crystallization and has less heavy metal contamination than other Zn sources such as zinc sulfate (ZnSO_4_) and ZnO. Diet supplemented with TBZC has low oxidation risk during storage due to the low hygroscopic capacity of TBZC [6]. It is reported that the relative Zn bioavailability in TBZC is one to two times higher than that of feed-graded ZnO [6,7]. As a result, the TBZC is an effective growth promoter requiring a lower dose concentration than that of ZnO and with less fecal Zn excreted into the environment. Furthermore, the TBZC has shown a stronger anti-diarrhea effect at a lower Zn dose compared to ZnO used in pharmacological doses in weaned pig diets, which is due to its better ability to reduce intestinal permeability and prevent the disruption of barrier integrity [1]. Therefore, TBZC would be a good source of inorganic Zn supplemented in weaned pig diets.

Previous research has shown that growth performance was no longer improved if the supplemental TBZC concentration was greater than 1,500 mg/kg in nursery pigs [7]. Hill et al [2] reported that this turning point for growth performance was 2,000 mg/kg for dietary ZnO supplementation in weaned pigs. Moreover, Swinkels et al [8] found that high concentrations of dietary Zn significantly increased pancreas and liver weights of pigs. The proteomic analysis also revealed that high concentrations of dietary Zn triggered many oxidative stress reactions in pancreases of young pigs [9]. All these results suggested that high concentration of dietary Zn is harmful to pig health and offsets its partial growth-promoting effect. However, few studies have focused on the feed safety, the appropriate dosage range and the Zn excretion of dietary TBZC supplementation in weaned pigs.

Therefore, the objectives of this study were i) to evaluate the effects of different concentrations of dietary supplemental TBZC on growth performance, organ morphology, hematological and serum biochemical characteristics in weaned pigs, then to determine dose-dependent effects of TBZC on response variables, and ii) to test whether replacing pharmacological concentrations of dietary ZnO with TBZC would further improve growth performance and decrease Zn excretion in weaned pigs.

## MATERIALS AND METHODS

All procedures used in this study were conducted in accordance with the Chinese Guidelines for Animal Welfare and approved by the China Agricultural University Institutional Animal Care and Use Committee (Beijing, China). Two experiments were conducted using 420 crossbred (Duroc× Landrace× Yorkshire) weaned pigs raised at the China Agriculture University Animal Experimental Base (Fengning, China). In both experiments, pigs were housed in 1.2×2.1 m^2^ pens equipped with plastic slatted floors, automatic stainless steel nipple drinkers, and feeders. Initial room temperature was maintained at 28°C and then decreased by 1°C weekly, and the relative humidity was controlled at 60% to 70%. All pigs had *ad libitum* access to water and feed throughout the experiment. The ZnO containing 75% Zn was a common feed-graded source and provided by Beijing Tonglixingke Company (Beijing, China), and the TBZC [Zn_5_(OH)_8_Cl_2_·H_2_O] containing 55% Zn was provided by Changsha Xingjia Biotechnology Company (Changsha, China).

### Animals, diets, and experimental design

In Exp. 1, one hundred and eighty crossbred weaned pigs (8.92±1.05 kg of body weight [BW] and 28±3 d of age) were randomly assigned to 5 dietary treatments in a completely randomized design for a 28-d experiment. There were 6 pens per treatment with 6 pigs (3 barrows and 3 gilts) per pen. Five dietary treatments were formulated using a two-phase feeding program, with the basal diet (Table 1) containing 125 mg/kg Zn (as-fed basis) as ZnSO_4_, or with 0, 1,200, 1,800, 2,400, or 3,000 mg/kg Zn (as-fed basis) as TBZC. All diets were fed in mash form and were formulated to meet or exceed the nutrient requirements of 7 to 11 and 11 to 25 kg pigs recommended by NRC [10].

In Exp. 2, two hundred and forty crossbred weaned pigs (7.66±1.09 kg of BW and 28±3 d of age) were randomly assigned to 5 dietary treatments in a completely randomized design for a 28-d experiment. Each treatment diet was fed to 8 replicated pens with 6 pigs (3 barrows and 3 gilts) per pen. The basal diets used in Exp. 2 (Table 1) were formulated to meet or exceed the nutrient requirement, except for Zn, of 7 to 11 and 11 to 25 kg pigs recommended by NRC [10]. No supplemental Zn source was included in the basal diet other than the intrinsic Zn in the other ingredients. The 5 dietary treatments consisted of a negative control diet without Zn addition (NC), a positive control diet with ZnO supplementation (NC+2,250 mg/kg Zn as ZnO), and 3 experimental diets supplemented with different concentrations of TBZC (NC+1,000, 1,250, and 1,500 mg/kg Zn as TBZC).

### Sample collection

In both experiments, BWs and feed consumption (on pen basis) were measured on d 0, 14, and 28 to calculate the average daily gain (ADG), average daily feed intake (ADFI), and feed efficiency (G:F).

Diarrhea rate was calculated as:

$$\begin{array}{l} \text{Diarrhea rate }(\%)=(\text{number of pigs with diarrhea}\times \text{diarrhea days})\\ /(\text{number of pigs}\times \text{total observational days})\times 100. \end{array}$$

In Exp. 1, one pig with median BW from each pen was selected to collect blood samples via the anterior vena cava on d 28 after an overnight fasting. Two blood samples were collected for each pig: one into 10-mL sodium-heparinized vacutainer tubes, and then immediately placed on ice to measure hematological parameters; another into 10-mL vacutainer tubes without anticoagulant. Subsequently, serum from the second blood sample was separated by centrifuged (Heraeus Biofuge 22R Centrifuge, Hanau, Germany) at 3,000×g for 10 min at 4°C and stored at −20°C until further analysis. Pigs selected for blood sample collection were euthanized in the morning on d 29. Heart, liver, spleen, lung, and kidney were quickly removed and washed thoroughly with normal saline to remove the residual blood and then weighed. The tissue samples from these organs were immediately fixed in 2.5% (v/v) glutaraldehyde-polyoxymethylene solution for morphological analysis. Tissues of heart, liver, kidney, and pancreas were sampled and stored at −70°C for minerals analysis. Liver tissue from one lobe was sampled and immediately stored in liquid nitrogen for antioxidant parameter analysis.

In Exp. 2, approximately 200 g of fecal samples were col lected from each pen for 3 days (from d 26 to 28) and were stored at −20°C. After thawing, the 3 days collection of fecal samples were pooled by pen and then dried at 65°C for 72 h. All samples were ground to pass through a 1-mm screen (40 mesh) before analysis. Chromic oxide (0.3%) was used as an indigestible indicator.

### Mineral analysis

Feeds (Exp. 1 and Exp. 2), organs (Exp. 1) and fecal (Exp. 2) samples were wet-digested using nitric-perchloric acid (3:1) and then diluted with deionized distilled water for analyses of minerals. Concentrations of Zn, copper (Cu), and iron (Fe) were analyzed using a Flame Atomic Absorption Spectrophotometry (Z-5000; Hitachi, Tokyo, Japan). The percentages of mineral absorption were determined using the indicator method.

The following equations were used to calculate the per centages of mineral absorption, average daily mineral intake and average daily fecal mineral content:

$$\begin{array}{l} \text{PM}=1-(\text{DC}\times \text{FM})/(\text{FC}\times \text{DM})\\ \text{Average daily mineral intake}=\text{ADFI}\times \text{DM}\\ \text{Average daily fecal mineral}=\text{ADFI}\times \text{DM}\times (1-\text{PM}) \end{array}$$

Where DC is the content of Cr _2_O_3_ in the diets, FM is the content of mineral in the feces, FC is the content of Cr_2_O_3_ in the feces, DM is the content of mineral in the diets, ADFI is the average daily feed intake (d 26 to 28; as-fed basis) and PM is the percentages of mineral absorption.

### Hematological parameters, serum biochemistry and liver antioxidant parameters

For hematological analysis, levels of hemoglobin (HGB) and hematocrit (HCT), and platelet (PLT), total white blood cells (WBC) and red blood cells (RBC) counts in blood samples were determined using a full automated hematology analyzer (MindrayBC-2800, Shenzhen, China). The concentrations of aspartate aminotransferase (AST), alanine aminotransferase (ALT), total bilirubin (T-BIL), creatinine (CREA), alkaline phosphatase (ALP), glucose (GLU), total protein (TP), albumin (ALB) and urea nitrogen in serum (SUN) were measured by corresponding commercial kits (BioSino Bio-technology and Science Inc., Beijing, China) using an automatic biochemical analyzer (Hitachi 7020, Hitachi High-Technologies Corporation, Japan). Concentration of malondialdehyde (MDA), total antioxidant capacity (T-AOC) and superoxide dismutase (SOD) and glutathione peroxidase (GSH-Px) activities in liver were determined using the corresponding commercial assay kits (Nanjing Jiancheng Bioengineering Institute, Nanjing, China) followed the manufacturer’s instructions.

### Organ indexes and histology

Indexes (the ratio of the organ weight to BW) of heart, liver, spleen, lung, and kidney of piglets slaughtered were calculated. The histological analyses of heart, liver, spleen, lung, and kidney were measured according to the method provided by Kuang et al [11]. Changes in organic structure were visualized using a light microscope. All organ samples were also examined for histological evidence of abnormality by an experienced technician.

### Statistical analysis

Data for both experiments were checked for normality and outliers using the UNIVERIATE procedure of SAS 9.4 (SAS Institute Inc., Cary, NC, USA). Data were then analyzed through one-way analysis of variance using the general linear model procedure of SAS. Pen served as the experimental unit for growth performance, diarrhea incidence, and mineral excretion data. Individual pig was considered as the experimental unit for other indexes. Treatment means were calculated using the LSMEANS statement and separated by the Student-Newman-Keuls test. In addition, coefficients for unequally spaced contrasts were generated by interactive matrix language procedure of SAS, then orthogonal polynomials contrasts were conducted using the CONTRAST statement to determine linear and quadratic effects of increasing dietary concentrations of supplemental TBZC. Moreover, CONTRAST statement was also used to compare the effects of the NC diet vs the ZnO diet, and the ZnO diet vs mean of TBZC treatments on growth performance and diarrhea incidence, and the effects of 1,000 mg/kg, 1,250 mg/kg, or 1,500 mg/kg TBZC diet vs the ZnO diet on mineral absorption and excretion of weaned pigs in Exp. 2. Differences were declared significant at p<0.05, while 0.05≤p<0.10 was considered to indicate a trend in the data.

## RESULTS

The mineral composition of diets from the laboratory analysis are presented in Tables 2 and 3 for Exp. 1 and 2, respectively. The results showed that the dietary Zn concentrations were close to the formulated values.

### Growth performance and diarrhea incidence in Exp. 1

In Exp. 1, in phase 1 (d 0 to 14), weaned pigs fed diets containing TBZC showed greater ADG (p<0.05) than pigs fed the basal diet, and there was no further increase in ADG when dietary Zn concentration as TBZC increased from 1,200 to 3,000 mg/kg (Table 4). In phase 2 (d 14 to 28) and during the overall 28-day period, the ADG (p<0.01) and ADFI (p<0.05) of weaned pigs responded quadratically as the dietary TBZC concentration increased, with pigs fed 1,800 mg/kg Zn as TBZC having the greatest ADG numerically. However, pigs fed 3,000 mg/kg Zn as TBZC did not perform differently compared with pigs fed the basal diet.

In either phase 1 or 2 or during the whole period, the G:F and diarrhea incidence responded quadratically (p<0.01) as the dietary TBZC concentration increased. The G:F reached numerically the greatest in pigs fed diet with 1,800 mg/kg Zn as TBZC. In addition, pigs fed diets containing TBZC significantly reduced the diarrhea incidence (p<0.05) compared with pigs fed the basal diet.

### Hematological parameters in Exp. 1

On d 28, the HGB level (p<0.01) and HCT (p<0.05) increased linearly as TBZC supplemental concentration increased (Table 5). Weaned pigs fed 3,000 mg/kg Zn as TBZC showed greater RBC count (p<0.01) than pigs fed the basal diet. However, no significant differences (p>0.05) of WBC and PLT among the treatment groups were observed.

### Serum biochemical and liver antioxidant parameters in Exp. 1

The AST and TP concentrations in serum of weaned pigs on d 28 changed quadratically (p<0.01 and p = 0.04, respectively) as the incremental concentrations of TBZC supplemented in diets (Table 6). Dietary TBZC inclusion linearly increased (p<0.01) ALT and ALP levels in serum, and weaned pigs fed 2,400 mg/kg Zn as TBZC had the greatest ALT and ALP concentrations numerically. Additionally, there were no significant differences on T-BIL, CREA, GLU, ALB, and SUN levels in serum among all the treatment groups (date not shown).

In liver, the GSH-Px and T-AOC activities quadratically increased (p<0.01) as the TBZC supplemental concentration increased, and pigs fed 1,800 mg/kg Zn as TBZC had the greatest (p<0.01; Table 6) GSH-Px activities. Hepatic MDA content on d 28 showed a quadratic pattern (p<0.01) as TBZC supplemental concentration increased. In addition, pigs fed 3,000 mg/kg Zn as TBZC showed the lowest GSH-Px and T-AOC activities, as well as the highest MDA content (p<0.01).

### Organ weight and organ Zn concentrations in Exp. 1

Incremental increase in TBZC supplemental concentration linearly increased (p<0.05) liver weight, liver index and kidney index, and quadratically altered lung weight, kidney weight (p<0.05) and lung index (p<0.01) in weaned pigs on d 28 (Table 7). However, there were no significant effect of dietary TBZC supplementation on weights and indexes of heart and spleen in weaned pigs (p>0.05).

On d 28, increment in TBZC supplemental concentration linearly increased (p<0.01) Zn concentrations in heart, liver, kidney and pancreas (Table 8). The Zn concentrations of heart, liver and kidney in pigs fed diets containing 2,400 or 3,000 mg/kg Zn as TBZC were greater than those of pigs fed 1,200 mg/kg Zn as TBZC and the basal diets.

### Histopathological analysis of organs in Exp. 1

**Figure 1:**
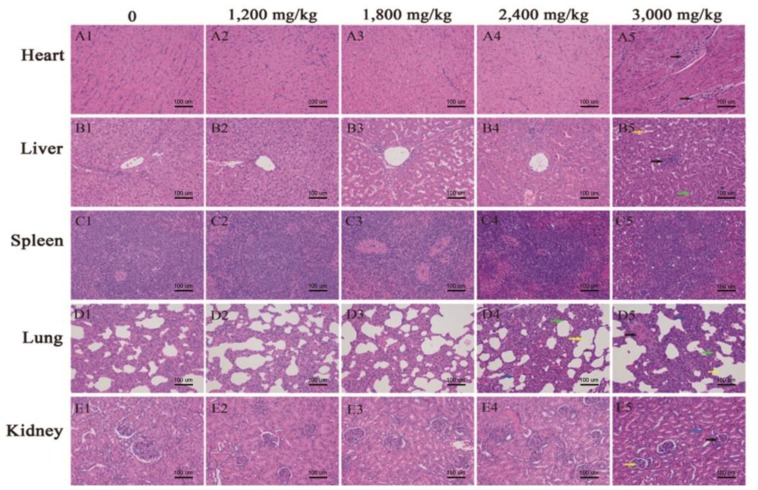
Effects of different concentrations (0 to 3,000 mg/kg zinc) of dietary tetrabasic zinc chloride (TBZC) supplementation on tissue structure of heart, liver, spleen, lung and kidney in weaned pigs. A–E represent tissues of heart, liver, spleen, lung and kidney, and 1–5 represent dietary TBZC supplemental concentrations of 0, 1,200, 1,800, 2,400, and 3,000 mg/kg zinc (Zn), respectively. Heart tissue with 3,000 mg/kg Zn as TBZC (A5) showed interstitial edema (black arrows); liver tissue with 3,000 mg/kg Zn as TBZC (B5) showed stem cell swelling (green arrow), hepatic sinus dilatation (yellow arrow) and lymph cell infiltration (black arrow); lung tissue with 2,400 and 3,000 mg/kg Zn as TBZC (D4 and D5, respectively) exhibited alveolar collapse and compensatory expansion of the alveolar spaces (yellow arrows), thickened alveolar wall (green arrows), and proteinaceous mucus in the alveolar space (blue arrows and black arrow); kidney tissue with 3,000 mg/kg Zn as TBZC (E5) exhibited glomerular lobes (yellow arrow), atrophy (black arrow), and tubulointerstitial hyperemia (blue arrow). H&E, ×200.

Morphological changes of heart, liver, spleen, lung, and kidney with graded concentrations of TBZC supplementation are shown in pictures A1–A5, B1–B5, C1–C5, D1–D5, and E1–E5, respectively (Figure 1). Histopathological examination revealed dose-related lesions in heart, liver, lung and kidney, and mild or severe histological lesions mainly focused on treatments with 2,400 and 3,000 mg/kg Zn as TBZC. Briefly, interstitial edema was observed in heart (A5), hepatocytes swelled and lymphocytes infiltrated in liver (B5). Some alveolar collapsed and the alveolar space enlarged compensatory. In addition, visible protein mucus appeared in the alveolar space in treatments with 2,400 and 3,000 mg/kg Zn as TBZC (D4 and D5). In kidney, glomerular atrophy and renal tubular interstitial hyperemia were found (E5).

### Growth performance and diarrhea incidence in Exp. 2

In phase 1 (d 0 to 14) and during the overall 28-d period, pigs fed 2,250 mg/kg Zn as ZnO had greater ADG (p<0.05) than pigs fed the NC diet, but lower G:F (p<0.05) than pigs receiving Zn from TBZC, regardless of TBZC supplemented concentrations (Table 9). In phase 2 (d 14 to 28), pigs fed 2,250 mg/kg Zn as ZnO showed greater G:F (p<0.05) than pigs fed the NC diet, but lower ADG (p<0.05) compared with pigs fed the TBZC supplemented diets.

During each phase in Exp. 2, ADG and G:F responded lin early (p<0.01) as the dietary supplemental TBZC concentration increased (Table 9). Weaned pigs fed 2,250 mg/kg Zn as ZnO had lower (p<0.01) diarrhea incidence than pigs fed the NC diet and showed similar diarrhea-reducing effect compared with pigs fed the TBZC supplemented diets.

### Mineral absorption and excretion in Exp. 2

The percentages of Zn and Cu absorption responded quadratically (p<0.01) with increased dietary TBZC concentrations, and there was a quadratically response tendency (p = 0.06) for the percentages of Fe absorption (Table 10). Weaned pigs fed 1,000 mg/kg Zn as TBZC had lower (p<0.01) fecal Zn and greater (p = 0.02) Cu absorption than pigs fed the ZnO diet. The percentages of Zn, Cu, and Fe absorption in pigs fed diets containing 1,250 mg/kg Zn as TBZC were greater than those of pigs fed the ZnO diet (p<0.01). However, pigs fed 1,500 mg/kg Zn as TBZC showed negative Zn absorption on d 28.

## DISCUSSION

Previous studies have shown that the growth performance of weaned pigs quadratically enhanced as the dietary supplemental TBZC concentration increased [6,7]. Similarly, our study (Exp. 1) also showed that the growth performance quadratically changed as TBZC supplementation concentration increased from 0 to 3,000 mg/kg for the overall 28-d period. However, in phase 2 and overall period, the growth performance of weaned pigs decreased to the same level as the control group when TBZC was supplemented at 3,000 mg/kg, which is consistent with previous reports that pigs fed 3,000 mg/kg Zn had similar or slightly increased growth performance compared to the Zn-deficient treatment [3,6,12]. Growth retardation under excessive ingestion of Zn were also observed in rats and hens [13,14]. These results indicated that long-term intake of high-Zn diets may have potentially negative effects on growth and G:F in animals such as weaned pigs. The impaired growth performance was most likely due to a marginal Zn toxicity as Zn accumulates in tissues. Moreover, 1,800 mg/kg Zn as TBZC supplementation resulted in the best growth performance in phase 2 and the overall period, while 1,200 mg/kg Zn as TBZC led to the best growth performance in phase 1. Mavromichalis et al [7] reported that concentrations of supplemental TBZC greater than 1,500 mg/kg were no more efficacious on growth performance than the 1,500 mg/kg Zn dose concentration on nursery pigs in a 21-d assay, which exactly falls into our optimal concentration range (1,200 to 1,800 mg/kg Zn) of dietary TBZC supplementation in weaned pigs from Exp. 1.

In practice, pharmacological dose of Zn is widely supple mented in weaned pig diets to enhance the growth performance and, more importantly, to reduce the incidence of post-weaning diarrhea, which has been widely reported [6,15]. In our study, pigs fed diets supplemented with TBZC showed significantly lower diarrhea incidence than those fed the basal diet, indicating that TBZC is an effective Zn source in diarrhea alleviation, which is consistent with another study focusing on dietary TBZC supplementation and fecal scores [6]. However, the mechanisms of the anti-diarrhea effect by Zn are still not fully elucidated. It had been suggested that this effect of Zn could be attributed to its function in inhibiting pathogenic bacterial adhesion, preserving intestinal epithelial barrier integrity, and enhancing mucosal restorability [16]. Zhang and Guo [1] reported that dietary Zn supplementation at 2,000 mg/kg led to a marked decline in fecal scores caused by reduced intestinal permeability. Katouli et al [17] pointed out that Zn included in the weaned pig diets could maintain the stability of intestinal microecology, and thereby reduce diarrhea occurrence caused by the excessive proliferation of pathogenic *Escherichia coli* (*E. coli*). Similarly, sequencing studies in pigs have shown that high dietary Zn supplementation increased intestinal microflora diversity and reduced the occurrence of pathogenic *E. coli* strains [18]. Therefore, an improved gut health through Zn supplementation may explain the alleviated diarrhea. The reduction in chloride secretion and subsequent reduction of water secretion into the intestinal may also contribute to diarrhea reduction [19]. Recent studies have reported a beneficial effect of nano-size Zn or organic Zn supplementation on immune response [5], during which process Zn could play a role through more complex mechanisms. Generally, weaned pigs with diarrhea are often accompanied by lower growth rate and feed utilization. Therefore, dietary Zn supplementation reduced the diarrhea incidence, and also could lead to the improved growth performance. Our data demonstrated that TBZC as a Zn source has a strong anti-diarrhea effect even at the dose of 1,200 mg/kg Zn, indicating that TBZC has a potential to replace ZnO as an anti-diarrhea agent.

Hematological traits are important parameters in evaluat ing health and immunity status of individual animal. In our study, the RBC count, HGB concentrations and HCT increased as the incremental concentrations of dietary TBZC supplementation, which may support the previous findings that Zn has a crucial effect on DNA synthesis, cell division and protein synthesis [20]. Study on rats supplied with low-zinc diet showed a significant reduction in RBC count, indicating that Zn is involved in erythrocyte formation and maturation [21]. Yanagisawa et al [13] reported that serum erythropoietin, the essential growth factor for erythroid lineage cell proliferation and differentiation, was profoundly increased in high-Zn diet group. Therefore, Zn may increase the RBC count by up-regulating the serum erythropoietin. Our results further confirmed the previous report that dietary Zn is involved in an increased HGB content and HCT in pigs [22], and these increases are obviously caused by the elevated RBC count. No effects of Zn sources supplementation on WBC and PLT levels were observed, which is consistent with previous studies [15,22].

Increased ALT and AST concentrations in serum are com monly used as diagnostic indicators for liver damage and dysfunction. Serumal ALP is a sensitive biomarker of hepatocyte necrosis by biliary obstruction. As shown in our study, AST and ALT levels in serum increased sharply when pigs were fed diets containing more than 2,400 mg/kg Zn as TBZC, and similar changes of transaminases were also observed in rats [11]. Moreover, the ALP activity elevated linearly with the gradual increased supplemental concentrations of Zn sulfate or organic Zn in previous studies [23,24], which is in accordance with our results. The high serumal ALP activity together with the transaminases indicates a possible damage in liver in pigs with high concentrations of TBZC intake.

Zinc plays an important role in maintaining the antioxi dant enzyme activities in tissues, protecting the cells against oxidative stress, and scavenging the free radicals [20,25,26]. The Zn-related antioxidant enzymes, including GSH-Px and SOD, could limit the effects of reactive oxygen species on tissues and are active in defense against oxidative cell damage [25]. In our study, 1,200 or 1,800 mg/kg Zn as TBZC supplementation in diets increased the activities of GSH-PX and T-AOC in liver, which is in accordance with other reports that dietary supplementation with appropriate concentrations of Zn could improve liver antioxidant capacity and help maintain liver health [14,26]. Moreover, it was revealed in our study that the activities of these antioxidant enzymes would decrease when supplementation concentration of dietary TBZC exceeded 1,800 mg/kg Zn. On the other hand, the MDA content showed opposite change with the aforementioned antioxidant enzyme activities. The MDA is a critical indicator for lipid peroxidation and oxidative damage caused by reactive oxygen species, which would influence cell membrane fluidity as well as the integrity of biomolecules [25,26]. Increased MDA content caused by high dietary Zn concentration is reported to be associated with lower antioxidant enzyme activities, and also associated with the oxidative cell damage in liver, which would allow ALT and AST to permeate into blood due to increased cell membrane permeability [25]. Therefore, the increased MDA level is in agreement with the increased transaminase levels in serum in our study. In general, the above results demonstrated that an appropriate concentration of dietary TBZC supplementation could enhance the hepatic antioxidant capacity in weaned pigs, while high TBZC concentration such as 2,400 mg/kg Zn may result in oxidative damage in liver, thus may negatively affect the growth performance of pigs.

Analysis of organ weight is a vital endpoint for identifi cation of potentially harmful effects of experimental additives in animals. The linear increase in liver and kidney weights in our study further confirmed the previous results that high dietary Zn concentration led to the enlargement of liver and kidney weights [20,27]. The enlargement of organ weights is associated with the toxicity generated from excess Zn accumulation in tissues [28], which has been proved by our observations that feeding 3,000 mg/kg supplemental Zn as TBZC resulted in a 9.3- and 10.6-fold increases in amount of Zn accumulation in liver (106.6 vs 987.3 mg/kg) and kidney (22.6 vs 240.4 mg/kg), respectively, compared with pigs fed the basal diet. Furthermore, increased synthesis of the Zn storage protein metallothionein and the oxidative stress-related proteins under high Zn supplying may also contribute to the heavier liver weight [27,28]. It has been reported that increased kidney weight may be related to edema, and one possible pathogenesis of renal lesions could be increased concentration of the supplemented test substance (or its metabolite) as a function of the renal osmotic concentration gradients [29], which is consistent with the severe injury in the morphology of kidney in our study. However, the weight and index of lung decreased when TBZC supplementation increased from 1,800 mg/kg Zn to 2,400 mg/kg Zn, indicating that lung may be more sensitive to the excess Zn concentration. Further studies are necessary to elucidate the mechanisms of organ damage with high-dose of Zn supplementation.

In the present study, the morphology of organs was evaluat ed based on histopathological observations. Zinc accumulation in heart, liver, lung and kidney resulted in mild or moderate structural changes to the aforementioned organs, and the damage of organs occurred when pigs were fed more than 2,400 mg/kg Zn as TBZC, which is in accordance with the results from the hematological and serum biomarkers analysis. Additionally, other researchers have observed similar or more pronounced changes in the hepatic and splenic tissues under high concentrations of Zn [11,13].

Based on the results of Exp. 1, we can conclude that dietary TBZC supplementation at 1,200 to 1,800 mg/kg Zn resulted in the best growth performance and health status in weaned pigs. However, weaned pigs fed 1,800 mg/kg Zn as TBZC showed a negative Zn balance, with Zn excreting in feces exceeding Zn consumed on a daily basis (data not shown). Hence, Exp. 2 was conducted with three concentrations of TBZC near 1,200 mg/kg Zn addition to determine the optimal TBZC supplemental concentration in weaned pigs to minimize the fecal Zn excretion, and ZnO was a positive control in this experiment.

Our results in Exp. 2 further confirmed that feeding phar macological concentrations of ZnO could increase growth performance and reduce diarrhea incidence in weaned pigs. Furthermore, compared to dietary supplementation with 2,250 mg/kg Zn as ZnO, TBZC showed a strong growth-promoting effect and a similar anti-diarrhea effect at lower doses, suggesting that TBZC may have higher bioavailability than ZnO. Mavromichalis et al [7] reported that the bioavailability of Zn in TBZC was two times more than that in ZnO. Zhang and Guo [6] reported that the bioavailability of Zn in TBZC was 122.1% to 159.3% relative to that in ZnO based on different response characteristics. This may be attributed to the ionic form of Zn in TBZC being more easily absorbed in intestine than the Zn in the covalent bond form in ZnO, since Zn is transported through the enterocytes by specific Zn transporters in the ionic form. Moreover, the high bioavailability of Zn could greatly help to decrease the Zn excretion.

In Exp. 2, after 28-d trial, weaned pigs fed the NC diet were in a negative Zn balance, indicating that part of the endogenous Zn is lost from the intestine. Endogenous Zn excretion is an unavoidable and constant metabolic loss, mainly being excreted by the secretion of pancreatic juice and intestinal exfoliated cells [30]. The Zn absorption in percentage was greater in pigs fed 1,000 or 1,250 mg/kg Zn as TBZC than those fed 2,250 mg/kg Zn as ZnO, which further demonstrated that dietary TBZC supplementation as a Zn source could reduce Zn excretion. When pigs were fed 1,500 mg/kg Zn as TBZC the quantity of Zn in feces was approximately equal to the sum of dietary Zn intake and endogenous Zn excretion, and the Zn absorption seemed to reach a saturation point, indicating that this high concentration of TBZC was slightly excessive for weaned pigs. On the other hand, the results on the saturation point illustrated that pigs had absorbed excess Zn during d 26 to 28, which also proved that TBZC had greater bioavailability than ZnO during this period. Therefore, the optimal dose for dietary TBZC supplementation to reduce Zn excretion is 1,000 to 1,250 mg Zn/kg. Moreover, the percentages of Cu and Fe absorption rose before the supplemented TBZC concentration reached 1,250 mg/kg Zn. At lower dietary Zn concentrations, increased Cu and Fe absorption could be related to the Zn concentration which plays a role in the improved intestinal structure and growth performance [1]. Subsequently, reduction in Cu and Fe absorption after 1,250 mg/kg Zn as TBZC supplementation may be due to the antagonistic action of high Zn concentrations in the Cu and Fe absorption process, which has been reported by previous studies [3,30].

## CONCLUSION

Overall, our study demonstrated that dietary TBZC supplementation at 1,000 to 1,800 mg/kg Zn improved growth performance, alleviated the diarrhea incidence and had no deleterious effect in weaned pigs. When dietary TBZC supplementation concentration was below 1,250 mg/kg Zn, the Zn excretion in manure could be significantly reduced compared to a pharmacological concentration of ZnO. However, excessive (more than 2,400 mg/kg Zn) dietary TBZC inclusion had adverse effects on organ tissue of weaned pigs. Therefore, TBZC could be a potential effective alternative to ZnO and the recommended supplemental concentration of TBZC in weaned pig diets is 1,000 to 1,250 mg/kg Zn.

## Figures and Tables

**Table 1:** Ingredients and chemical compositions of the basal diets used in Exp. 1 and 2 (as-fed basis)

Items	Exp. 1		Exp. 2	
	
	d 0 to 14	d 14 to 28	d 0 to 14	d 14 to 28
Ingredient (%)				
Corn	60.58	66.16	51.64	58.53
Soybean meal (44% CP)	10.00	17.00	16.85	13.40
Extruded soybean	-	-	14.20	11.00
Soy protein concentrate	9.00	5.60	-	-
Fish meal	4.70	2.00	4.00	5.00
Whey power	10.00	4.00	8.00	7.00
Soybean oil	1.26	1.00	1.65	1.32
Dicalcium phosphate	0.79	0.86	1.21	1.10
Sodium bicarbonate	0.88	0.85	-	-
Limestone	0.74	0.73	0.74	0.50
Salt	0.30	0.30	0.30	0.50
L-Lys HCl	0.60	0.50	0.46	0.40
L-Thr	0.22	0.17	0.13	0.10
DL-Met	0.23	0.17	0.12	0.15
Chromic oxide	-	-	-	0.30
Choline chloride (40% Choline)	0.20	0.16	0.20	0.20
Vitamin-mineral premix1)	0.50	0.50	0.50	0.50
Total	100.00	100.00	100.00	100.00
Calculated composition (%)				
Digestible energy (kcal/kg)	3,542	3,480	3,550	3,480
Crude protein	20.50	19.00	20.50	18.90
Ca	0.84	0.74	0.85	0.75
P	0.67	0.64	0.66	0.65
Available P	0.47	0.44	0.46	0.44
Standardized ileal digestible amino acid (%)				
Lys	1.42	1.30	1.41	1.30
Met	0.41	0.40	0.41	0.40
Thr	0.82	0.75	0.80	0.74
Trp	0.22	0.21	0.22	0.21

1) Vitamin and mineral premix provided the following per kilogram of diet: 12,000 IU vitamin A (vitamin A acetate), 2,500 IU vitamin D (vitamin D_3_), 30 IU vitamin E (dl-α-tocopheryl acetate), 12 μg vitamin B_12_, 3 mg vitamin K (menadione sodium bisulfate), 15 mg d-pantothenic acid (calcium pantothenate), 40 mg nicotinic acid, 30 mg Mn (manganese oxide), 120 mg Fe (ferrus sulfate), 150 mg Cu (copper sulfate), 0.35 mg I (ethylenediamine dihydroiodide), and 0.3 mg Se (sodium selenite). In addition, vitamin and mineral premix used in Exp. 1 contained 125 mg Zn (zinc sulfate), and the premix in Exp. 2 did not.

**Table 2:** Analyzed mineral compositions of diets used in Exp. 1

Items	TBZC (mg/kg Zn)				

	0	1,200	1,800	2,400	3,000
Phase 1, d 0 to 14					
Zn (mg/kg)	142	1,340	1,942	2,539	3,144
Cu (mg/kg)	163	161	164	159	161
Fe (mg/kg)	312	323	318	318	319
Phase 2, d 14 to 28					
Zn (mg/kg)	144	1,346	1,947	2,546	3,143
Cu (mg/kg)	159	160	158	160	159
Fe (mg/kg)	322	327	330	323	325

Analysis conducted in duplicates, and the Zn concentration in the basal diet (no TBZC supplementation) was 125 mg/kg as zinc sulfate (ZnSO_4_).

TBZC, tetrabasic zinc chloride; Zn, zinc; Cu, copper; Fe, iron.

**Table 3:** Analyzed mineral compositions of diets used in Exp. 2

Items	TBZC (mg/kg Zn)				ZnO (mg/kg Zn)
	
	0 (NC)	1,000	1,250	1,500	2,250
Phase 1, d 0 to 14					
Zn (mg/kg)	33	1,045	1,272	1,568	2,259
Cu (mg/kg)	177	179	173	171	175
Fe (mg/kg)	288	293	290	281	280
Phase 2, d 14 to 28					
Zn (mg/kg)	29	1,072	1,315	1,580	2,149
Cu (mg/kg)	175	177	172	174	172
Fe (mg/kg)	298	294	295	285	277

Analysis conducted in duplicates.

TBZC, tetrabasic zinc chloride; NC, negative control without Zn source supplementation; ZnO, positive control, NC+2,250 mg/kg Zn as Zn oxide (ZnO); Zn, zinc; Cu, copper; Fe, iron.

**Table 4:** Effects of dietary tetrabasic zinc chloride supplementation on growth performance and diarrhea incidence of weaned pigs in Exp. 1

Items	TBZC (mg/kg Zn)					SEM	p-value		
	
	0	1,200	1,800	2,400	3,000		Treatment	Linear1)	Quadratic2)
Phase 1, d 0 to 14									
ADG (g)	331^b^	368^a^	367^a^	364^a^	366^a^	11.56	0.02	0.07	0.09
ADFI (g)	527	560	546	559	555	13.33	0.16	0.06	0.23
G:F	0.63^c^	0.66^ab^	0.67^a^	0.65^b^	0.66^ab^	0.01	<0.01	<0.01	<0.01
Diarrhea incidence (%)	10.95^a^	0.48^b^	0.71^b^	1.67^b^	0.95^b^	0.94	<0.01	<0.01	<0.01
Phase 2, d 14 to 28									
ADG (g)	387^bc^	417^ab^	438^a^	426^ab^	375^c^	15.24	<0.01	0.89	<0.01
ADFI (g)	654	689	691	690	659	15.54	0.28	0.58	0.04
G:F	0.59^c^	0.61^bc^	0.63^a^	0.62^ab^	0.57^d^	0.01	<0.01	0.41	<0.01
Diarrhea incidence (%)	6.91^a^	0.71^b^	0.24^b^	0.48^b^	0.95^b^	0.76	<0.01	<0.01	<0.01
Overall, d 0 to 28									
ADG (g)	359^b^	393^a^	403^a^	395^a^	370^b^	12.10	<0.01	0.04	<0.01
ADFI (g)	591	625	618	625	606	13.47	0.08	0.17	0.02
G:F	0.61^b^	0.63^ab^	0.65^a^	0.63^ab^	0.61^b^	0.01	0.04	0.37	<0.01
Diarrhea incidence (%)	8.93^a^	0.60^b^	0.48^b^	1.07^b^	0.95^b^	0.63	<0.01	0.03	<0.01

Values are least square means (n = 6 per treatment), and zinc (Zn) concentration in the basal diet (no TBZC supplementation) was 125 mg/kg as zinc sulfate (ZnSO_4_).

TBZC, tetrabasic zinc chloride; SEM, standard error of the mean; ADG, average daily gain; ADFI, average daily feed intake; G:F, feed efficiency.

1), 2) Linear and quadratic effects of increasing TBZC concentrations (0 to 3,000 mg/kg Zn).

a–d Least square means within a row with different superscripts differ (p<0.05).

**Table 5:** Effects of dietary tetrabasic zinc chloride supplementation on hematological parameters in weaned pigs in Exp. 1

Items	TBZC (g/kg Zn)					SEM	p-value		
	
	0	1,200	1,800	2,400	3,000		Treatment	Linear1)	Quadratic2)
White blood cell (×10^9^/L)	25.70	25.42	21.98	24.40	26.23	1.86	0.53	0.90	0.24
Red blood cell (×10^12^/L)	6.48^b^	6.34^b^	6.49^b^	6.80^ab^	7.09^a^	0.13	<0.01	<0.01	0.02
Hemoglobin (g/L)	98.19	105.40	102.25	113.60	114.40	4.25	0.06	<0.01	0.65
Hematocrit (%)	34.01	35.98	36.33	38.22	38.70	1.32	0.14	0.01	0.92
Platelet count (×10^9^/L)	206.75	228.80	201.50	228.00	221.00	14.55	0.58	0.51	0.86

Values are least square means (n = 6 per treatment), and zinc (Zn) concentration in the basal diet (no TBZC supplementation) was 125 mg/kg as zinc sulfate (ZnSO_4_).

TBZC, tetrabasic zinc chloride; SEM, standard error of the mean.

1),2) Linear and quadratic effects of increasing TBZC concentrations (0 to 3,000 mg/kg Zn).

a,b Least square means within a row with different superscripts differ (p<0.05).

**Table 6:** Effects of dietary tetrabasic zinc chloride supplementation on serum biochemical and liver antioxidant parameters in weaned pigs in Exp. 1

Items	TBZC (mg/kg Zn)					SEM	p-value		
	
	0	1,200	1,800	2,400	3,000		Treatment	Linear1)	Quadratic2)
Serum biochemical parameters									
AST (U/L)	67.60^ab^	64.30^ab^	53.90^b^	80.63^a^	80.75^a^	4.67	<0.01	0.03	<0.01
ALT (U/L)	60.11^b^	71.20^ab^	81.88^ab^	94.55^a^	91.37^ab^	6.30	0.02	<0.01	0.26
ALP (U/L)	376.6^b^	467.3^ab^	556.9^ab^	764.4^a^	682.6^ab^	83.37	0.03	<0.01	0.99
TP (g/L)	60.01	63.42	65.26	62.50	57.56	1.73	0.19	0.47	0.04
Liver antioxidant parameters									
MDA (nmol/mg)	0.33^bc^	0.34^b^	0.30^c^	0.30^c^	0.38^a^	0.01	<0.01	0.06	<0.01
GSH-Px (U/mg)	41.86^b^	42.27^b^	57.87^a^	48.38^b^	25.00^c^	2.26	<0.01	0.02	<0.01
T-AOC (U/mg)	0.64^b^	0.56^b^	0.76^a^	0.75^a^	0.46^c^	0.03	<0.01	0.13	<0.01
SOD (U/mg)	4.23	4.03	5.06	4.43	3.94	0.34	0.20	0.96	0.19

Values are least square means (n = 6 per treatment), and zinc (Zn) concentration in the basal diet (no TBZC supplementation) was 125 mg/kg as zinc sulfate (ZnSO_4_).

TBZC, tetrabasic zinc chloride; SEM, standard error of the mean; AST, aspartate aminotransferase; ALT, alanine aminotransferase; ALP, alkaline phosphatase; TP, total protein; MDA, malondialdehyde; GSH-Px, glutathione peroxidase; T-AOC, total antioxidant capacity; SOD, superoxide dismutase.

1),2) Linear and quadratic effects of increasing TBZC concentrations (0 to 3,000 mg/kg Zn).

a–c Least square means within a row with different superscripts differ (p<0.05).

**Table 7:** Effects of dietary tetrabasic zinc chloride supplementation on organ weight and visceral index in weaned pigs in Exp. 1

Items	TBZC (mg/kg Zn)					SEM	p-value		
	
	0	1,200	1,800	2,400	3,000		Treatment	Linear1)	Quadratic2)
Weight (g)									
Heart	104.40	100.79	101.84	90.85	98.44	5.84	0.56	0.25	0.85
Liver	465.59	473.00	475.69	498.17	532.45	15.90	0.07	0.01	0.14
Spleen	46.80	45.69	47.16	41.80	49.98	3.67	0.64	0.89	0.47
Lung	225.33^b^	230.99^b^	286.27^a^	252.19^ab^	219.98^b^	12.25	0.01	0.56	0.01
Kidney	94.56^b^	97.98^b^	102.47^b^	106.43^b^	121.60^a^	3.77	<0.01	<0.01	0.05
Visceral index (g/kg)									
Heart	5.58	5.50	5.68	5.14	5.31	0.29	0.69	0.37	0.72
Liver	25.91	26.00	26.86	27.72	29.29	0.85	0.08	0.01	0.18
Spleen	2.40	2.50	2.66	2.38	2.73	0.18	0.60	0.35	0.93
Lung	12.25^b^	13.25^ab^	15.47^a^	12.85^ab^	11.82^b^	0.70	0.03	0.89	<0.01
Kidney	5.05^c^	5.24^c^	5.77^b^	5.97^b^	6.56^a^	0.15	<0.01	<0.01	0.06

Values are least square means (n = 6 per treatment), and zinc (Zn) concentration in the basal diet (no TBZC supplementation) was 125 mg/kg as ZnSO_4_.

TBZC, tetrabasic zinc chloride; SEM, standard error of the mean.

1),2) Linear and quadratic effects of increasing TBZC concentrations (0 to 3,000 mg/kg Zn).

a–c Least square means within a row with different superscripts differ (p<0.05).

**Table 8:** Effects of dietary tetrabasic zinc chloride supplementation on organ Zn concentrations in weaned pigs in Exp. 1

Items	TBZC (mg/kg Zn)					SEM	p-value		
	
	0	1,200	1,800	2,400	3,000		Treatment	Linear1)	Quadratic2)
Heart (mg/kg)	24.27^c^	56.90^c^	92.22^bc^	149.16^ab^	199.85^a^	23.20	<0.01	<0.01	0.14
Liver (mg/kg)	106.60^d^	245.29^c^	644.28^b^	736.66^b^	987.32^a^	43.96	<0.01	<0.01	0.03
Kindey (mg/kg)	22.64^c^	26.85^c^	49.44^c^	135.02^b^	240.40^a^	8.00	<0.01	<0.01	0.12
Pancreas (mg/kg)	43.43^c^	91.92^c^	469.60^b^	363.74^bc^	937.69^a^	96.97	<0.01	<0.01	0.02

Values are least square means (n = 6 per treatment), and zinc (Zn) concentration in the basal diet (no TBZC supplementation) was 125 mg/kg as ZnSO_4_.

TBZC, tetrabasic zinc chloride; SEM, standard error of the mean.

1),2) Linear and quadratic effects of increasing TBZC concentrations (0 to 3,000 mg/kg Zn).

a–d Least square means within a row with different superscripts differ (p<0.05).

**Table 9:** Effects of dietary tetrabasic zinc chloride supplementation on growth performance and diarrhea incidence of weaned pigs in Exp. 2

Items	TBZC (mg/kg Zn)				ZnO (mg/kg Zn)	SEM	p-value1)			

							NC vs ZnO	TBZC vs ZnO	TBZC	
		
	0 (NC)	1,000	1,250	1,500	2,250				Linear	Quadratic
Phase 1, d 0 to 14										
ADG (g)	326	356	378	370	356	8.28	0.02	0.04	<0.01	0.72
ADFI (g)	513	548	557	551	553	13.82	0.03	0.89	0.14	0.54
G:F	0.63	0.65	0.68	0.67	0.64	0.01	0.28	0.02	<0.01	0.57
Diarrhea incidence (%)	17.76	7.47	4.78	3.75	5.06	0.88	<0.01	0.78	<0.01	<0.01
Phase 2, d 14 to 28										
ADG (g)	469	517	529	540	502	12.66	0.08	0.02	<0.01	0.98
ADFI (g)	883	922	907	929	892	22.78	0.74	0.16	0.22	0.90
G:F	0.53	0.56	0.58	0.58	0.56	0.01	0.04	0.07	<0.01	0.96
Diarrhea incidence (%)	9.59	3.75	2.73	2.53	3.60	0.56	<0.01	0.43	<0.01	0.06
Overall, d 0 to 28										
ADG (g)	397	437	454	455	429	8.41	0.02	0.01	<0.01	0.84
ADFI (g)	698	735	732	740	723	15.19	0.19	0.33	0.07	0.71
G:F	0.58	0.60	0.63	0.62	0.60	0.01	0.07	0.02	<0.01	0.68
Diarrhea incidence (%)	13.67	5.61	3.76	3.14	4.33	0.53	<0.01	0.73	<0.01	<0.01

Values are least square means (n = 8 per treatment).

TBZC, tetrabasic zinc chloride; NC, negative control without zinc (Zn) source supplementation; ZnO, positive control, NC+2,250 mg/kg Zn as Zn oxide (ZnO); SEM, standard error of the mean; ADG, average daily gain; ADFI, average daily feed intake; G:F, feed efficiency.

1) Comparisons between the NC diet vs the ZnO diet, and the ZnO diet vs mean of TBZC treatments (1,000 to 1,500 mg/kg Zn) using contrast. Linear and quadratic effects of increasing TBZC concentrations (0 to 1,500 mg/kg Zn).

**Table 10:** Effects of dietary tetrabasic zinc chloride supplementation on mineral absorption and excretion in weaned pigs in Exp. 2

Items	TBZC (mg/kg Zn)				TBZC_1000_ vs ZnO	SEM	p-value1)				

							ZnO (mg/kg Zn)	TBZC_1250_ vs ZnO	TBZC_1500_ vs ZnO	TBZC	
		
	0 (NC)	1,000	1,250	1,500	2,250					Linear	Quadratic
ADFI (d 26 to 28; as-fed basis, g)	955	994	984	1,001	971	43.96	0.71	0.85	0.67	0.17	0.79
Zn											
Intake (mg/d)	28	1,066	1,295	1,581	2,087	67.00	<0.01	<0.01	0.02	<0.01	0.49
Fecal (mg/d)	44	897	941	1,618	1,788	61.45	<0.01	<0.01	0.17	<0.01	<0.01
Absorption (%)	−58.46	15.86	27.17	−2.74	14.29	6.87	0.64	<0.01	<0.01	0.08	<0.01
Cu											
Intake (mg/d)	168	185	169	181	167	7.79	0.11	0.87	0.28	0.46	0.69
Fecal (mg/d)	147	157	132	158	152	7.05	0.66	0.07	0.59	0.62	0.41
Absorption (%)	12.41	15.15	21.91	12.09	8.73	1.55	0.02	<0.01	0.11	0.10	0.01
Fe											
Intake (mg/d)	285	292	290	285	269	12.70	0.21	0.29	0.43	0.31	0.93
Fecal (mg/d)	281	290	262	286	279	13.44	0.58	0.03	0.72	0.11	0.39
Absorption (%)	1.36	0.47	9.71	−0.67	−3.16	2.34	0.34	<0.01	0.44	0.57	0.06

Values are least square means (n = 8 per treatment).

TBZC, tetrabasic zinc chloride; NC, negative control without zinc (Zn) source supplementation; ZnO, positive control, NC+2,250 mg/kg Zn as Zn oxide (ZnO); SEM, standard error of the mean; ADFI, average daily feed intake; Cu, copper; Fe, iron.

1) Comparisons between the TBZC_1000_ diet vs the ZnO diet, the TBZC_1250_ diet vs the ZnO diet, and the TBZC_1500_ diet vs the ZnO diet using contrast. Linear and quadratic effects of increasing TBZC concentrations (0 to 1,500 mg/kg Zn).
